# Generation and Evaluation of an African Swine Fever Virus Mutant with Deletion of the *CD2v* and *UK* Genes

**DOI:** 10.3390/vaccines8040763

**Published:** 2020-12-14

**Authors:** Teshale Teklue, Tao Wang, Yuzi Luo, Rongliang Hu, Yuan Sun, Hua-Ji Qiu

**Affiliations:** 1 State Key Laboratory of Veterinary Biotechnology, Harbin Veterinary Research Institute, Chinese Academy of Agricultural Sciences, Harbin 150069, China; teshale12@gmail.com (T.T.); wangtaocaas@163.com (T.W.); luoyuzi@caas.cn (Y.L.); 2 Institute of Military Veterinary Medicine, Academy of Military Medical Sciences, Changchun 130000, China; ronglianghu@hotmail.com

**Keywords:** African swine fever, live attenuated virus, safety, protective efficacy, pig

## Abstract

African swine fever (ASF) is a highly contagious and often lethal disease caused by African swine fever virus (ASFV). ASF emerged in China in August 2018 and has since rapidly spread into many areas of the country. The disease has caused a significant impact on China’s pig and related industries. A safe and effective vaccine is needed to prevent and control the disease. Several gene-deleted ASFVs have been reported; however, none of them is safe enough and commercially available. In this study, we report the generation of a double gene-deleted ASFV mutant, ASFV-SY18-∆CD2v/UK, from a highly virulent field strain ASFV-SY18 isolated in China. The results showed that ASFV-SY18-∆CD2v/UK lost hemadsorption properties, and the simultaneous deletion of the two genes did not significantly affect the in vitro replication of the virus in primary porcine alveolar macrophages. Furthermore, ASFV-SY18-∆CD2v/UK was attenuated in pigs. All the ASFV-SY18-∆CD2v/UK-inoculated pigs remained healthy, and none of them developed ASF-associated clinical signs. Additionally, the ASFV-SY18-∆CD2v/UK-infected pigs developed ASFV-specific antibodies, and no virus genome was detected in blood and nasal discharges at 21 and 28 days post-inoculation. More importantly, we found that all the pigs inoculated with 10^4^ TCID_50_ of ASFV-SY18-∆CD2v/UK were protected against the challenge with the parental ASFV-SY18. However, low-level ASFV DNA was detected in blood, nasal swabs, and lymphoid tissue after the challenge. The results demonstrate that ASFV-SY18-∆CD2v/UK is safe and able to elicit protective immune response in pigs and can be a potential vaccine candidate to control ASF.

## 1. Introduction

African swine fever (ASF) is a devastating hemorrhagic disease of pigs that results in severe economic losses to the pig industry [1]. The disease is caused by African swine fever virus (ASFV), the only DNA virus that replicates in and transmitted by arthropods [2,3,4]. ASFV is highly stable in the environment [5], exceptionally complex in structure and genetic makeup [3,6], with 24 genotypes in which only genotypes I and II are circulating in Europe and Asia [7,8,9,10,11]. ASFV encodes many proteins with unknown function and with no significant sequence similarity to other viruses or eukaryotic proteins [6,12]. During ASFV infection, a variety of viral proteins are expressed to counteract the host immune responses. Nevertheless, genes responsible for ASF clinical outcomes remained poorly understood [6,13,14]. Over the past decades ASF has been a classic problem to African countries, except Sardinia, Italy, since 1978 [15]. However, in 2007, a highly virulent strain of ASFV jumped from East Africa to Georgia [16] and rapidly invaded parts of the Russian Federation and the European Union member countries, including Estonia, Latvia, Lithuania, Poland, and the Czech Republic [11]. A combination of factors, such as globalization and fast transport system, favors ASF to widely spread and reach more pig producing countries and emerged in China in August 2018. Thereafter, the disease became the primary cause of pig losses, posing a significant concern to the global pig production [10,17]. Currently, 75% of the world pig production is under continued ASF pressure with less efficient mitigation approaches [18,19,20,21]; hence, development of a vaccine is a pressing need.

Vaccination represents a principal opportunity to control infectious diseases, together with other prevention strategies. Despite the intense efforts made so far, no ASF vaccine is commercially approved [22,23]. The classically inactivated virions confer no protection, and there had been limited success with subunit vaccines, and about 50% protection was reported with DNA vaccines [24,25,26,27]. Only live attenuated ASFV mutants, either naturally evolved or developed by serial passage of virus in cell culture or by genetic manipulation, provide reliable protection against homologous, but rarely against heterologous ASFV strains [12,22,23]. The live attenuated ASFV isolates generated through the empirical approaches are less likely to be the future vaccines due to the possibly undesired or unpredicted molecular mechanisms of attenuation. Several ASFV genes, such *as A238L, A179L, A224L*, and *multigene family (MGF) 360*, and *505* genes, were reported to be nonessential for ASFV in vitro replication but interfere with the innate immune response at various points. These genes were considered as target genes to generate gene-deleted mutant viruses as ASF vaccines [28,29,30,31]. MGFs, involved in immuno-evasion [32], are present in all virulent isolates and partially deleted in naturally attenuated ASFV isolates [33,34]. Recently, MGFs-deleted mutants were reported with decreased virulence but induced protective immunity against the parent ASFV [35]. Exceptionally, CD2v-deleted BA71, BA71ΔCD2v, was reported to induce both homologous and heterologous protection [36]. Despite these promising results, the virulence of a given ASFV gene seems to be strain-dependent, and deletion of orthologous genes has different outcomes in different isolates [31]. Moreover, there is a doubt on the safety of the live attenuated ASF vaccines. None of the genetically modified ASFVs is safe enough for field application, and non-reproducible outcomes were reported [35,36,37,38,39,40].

We considered that safe and efficacious live attenuated ASF vaccines can be achieved through serial deletions of virulence-associated genes. The CD2-like ASFV protein (CD2v) is a structural transmembrane glycoprotein that mediates hemadsorption [40,41]. The deletion of this gene altered the virulence of field ASFV isolates [36,39], and additionally, this CD2-like ASFV protein is altered in naturally attenuated isolates [34]. The *UK* gene is a unique ASFV encoded small protein in the right variable region of the genome. Zsak et al. [42] reported that the *UK* gene is a significant virulence determinant of ASFV in domestic pigs. Additionally, simultaneous deletion of the *9GL* and *UK* genes reported to improve the safety of live attenuated ASFV, suggesting that the *UK* gene is virulence determinant [38]. Therefore, the aim of this study was to generate a live attenuated vaccine (LAV) based on the ASFV-SY18 isolate in China through sequential deletion of distinct virulence-associated *CD2v* and *UK* genes, as well as to examine its safety and protection potential in pigs.

## 2. Materials and Methods

### 2.1. Cells and Viruses

A highly virulent genotype II ASFV isolated from China, designated as ASFV-SY18 [10], was used as a parent virus throughout the experiment and as a genetic background virus to generate target gene-deleted mutant virus. Virus stocks were propagated in primary porcine alveolar macrophages (AM), and stored in −80 °C. AM were collected from specific-pathogen-free (SPF) pigs and maintained in RPMI 1640 medium (Thermo-Fisher Scientific, Carlsbad, CA, USA) containing 10% fetal bovine serum (Gibco, Grand Island, NY, USA) and 2% antibiotics-antimycotics (10,000 IU/mL of penicillin, 10,000 µg/mL of streptomycin, and 25 µg/mL of Gibco amphotericin B) (Gibco, Grand Island, NY, USA) at 37 °C under 5% CO_2_ according to the established procedures [43].

### 2.2. Construction of Transfer Vectors

In order to generate a double gene-deleted ASFV by the mammalian homologous recombination system [44], two recombinant transfer vectors were constructed. The recombination transfer vectors contain a marker gene, and the upstream and downstream homologous arms flanking the target viral genomic region. The targets were *CD2v* [nucleotides (nt) 73,36974,451] and *UK* (nt 184,331-184,621) open reading frames (ORFs) of the ASFV-SY18 isolate. Based on these target genes, the transfer vectors were constructed to insert marker genes replacing the target genes according to the established protocols [36]. The primers used to construct transfer vectors were synthesized by Kumei Biotechnology Co., Ltd., Changchun, China (Table 1). Briefly, right and left arms (each about 1.2 kb) flanking each target gene were amplified by PCR. The amplified fragments were designed to contain restriction enzyme sites for ligation to the plasmid. The corresponding left and right flanking fragments of each gene were then assembled by overlapping PCR to contain either *EGFP* (to replace the *CD2v*) or *dsRed* (to replace the *UK* gene) marker in the middle under the control of the p72 promoter. pOK12 vector was linearized, and the cassettes were then ligated to get the recombinant transfer vectors pOK12-p72∆CD2v-EGFP and pOK12-p72∆UK-dsRed using the Vazyme ClonExpress II One Step Cloning kit (Vazyme Biotech Co. Ltd., Nanjing, China). These recombinant transfer vectors containing the desired subclones were finally selected by PCR and confirmed by sequencing.

### 2.3. Generation of ASFV-SY18-∆CD2v/UK

The recombinant viruses were generated by homologous recombination between parental ASFV genome and the transfer vectors (pOK12-p72∆CD2v-EGFP and pOK12-p72∆UK-dsRed), following transfection and infection of AM according to the previously described methods [38]. Briefly, monolayers of AM were seeded in 12-well plates and 12 h later the cells were transfected with 1.5 µg of transfer vector using 5 µL of H-extremeGene HP DNA transfection reagent (Roche Diagnostics GmbH, Mannheim, Germany) according to the manufacturer’s instructions. ASFV-SY18 was then infected into AM at 16 h post-transfection. The infected AM were incubated until the recombinant virus expressing EGFP/dsRed was observed by fluorescent microscope (Nikon TE2000-U, Tokyo, Japan). Recombinant viruses were collected and passaged several times in AM and purified further by limiting dilution. Through these procedures, a CD2v-deleted ASFV was first generated and purified. Then, the *UK* gene was deleted from the CD2v-deleted ASFV to get a double gene-deleted designed as ASFV-SY18-∆CD2v/UK.

### 2.4. Identification of ASFV-SY18-∆CD2v/UK

#### 2.4.1. PCR

The homogenous ASFV-SY18-∆CD2v/UK obtained through successive purifications was further identified by PCR. DNA was extracted from the ASFV-SY18-∆CD2v/UK-infected AM, and the deletion of the *CD2v* and *UK* genes were confirmed by PCR using the primers specifically targeting these genes (Table 1). The *p72* (*B646L*) gene was used as a control to detect ASFV genome.

#### 2.4.2. Hemadsorption (HAD) Assay

The CD2v protein mediates hemadsorption of ASFV around infected cells in the presence of porcine red blood cells, and the hemadsorption assay (HAD) was used to calculate the median tissue culture infective dose (TCID_50_) of wild-type ASFV [45,46]. In this study, it was also used to indirectly determine whether ASFV-SY18-∆CD2v/UK had lost this property. Therefore, 2 × 10^5^ AM were seeded in 96-well plates overnight and infected with the parent and the recombinant viruses for comparison. After 2 days, 2% porcine red blood cells in PBS were added and the HAD was observed for 5 days.

#### 2.4.3. Fluorescence Assay

The ASFV-SY18-∆CD2v/UK mutant developed in this study contains *EGFP* replacing the *CD2v* gene, and this fluorescence tag was used to detect the virus under fluorescent microscope (Nikon TE2000-U, Tokyo, Japan) using protocols described by Borca et al. [44]. The TCID_50_ for each sample was determined based on the EGFP fluorescence in the sample dilutions [44,45].

#### 2.4.4. In Vitro Growth Kinetics

To determine whether the deletion of the two genes affected the in vitro replication of the virus, the in vitro growth kinetics of ASFV-SY18-∆CD2v/UK and ASFV-SY18 was compared in AM. Briefly, monolayers of AM were prepared overnight in 24-well plates and infected at a multiplicity of infection (MOI) of 0.01 of both the parent and recombinant viruses. The infected cells were incubated at 37 °C under 5% CO_2_ to allow adsorption of the viruses to the cells and after 1 h the medium was changed. The infected AM were incubated for 2, 12, 24, 48, 72, 96, or 120 h at 37 °C under 5% CO_2_. The cells and supernatants were then collected and frozen at −80°C at the indicated time points. The virus titer of the wild-type (ASFV-SY18) and mutant (ASFV-SY18-∆CD2v/UK) at each time point was determined using HAD and fluorescence assays, respectively, from the thawed cells. The multistep growth curve was constructed based on the virus titer of each time point.

### 2.5. Animal Experiments

Animal experiments were conducted to examine whether the simultaneous deletion of the *CD2v* and *UK* genes reduced the virulence of the ASFV-SY18 in vivo and to evaluate if this mutant can induce protective immune response. The animal experiments were performed in high containment biosafety level 3 (BSL3) facility in the Institute of Military Veterinary Medicine, Changchun, in accordance with the protocols established by the World Organization for Animal Health (OIE) and the Ministry of Agriculture and Rural Affairs of the Chinese Government. The experimental designs and protocols used in this study were approved by the Institutional Animal Care and Use Committee (IACUC) of academy of military medical science (AMMS) (Project permit: IACUC of AMMS-11-2020-018). All procedures were conducted in compliance with the Guidelines for the Ethical Review of Laboratory Animal Welfare of China National Standard GB/T 35892-2018 [47]. All the animal experiments were performed by licensed veterinarians, and the experimental pigs were cared by qualified veterinary technicians and trained animal caretakers.

A total of 10 forty-day-old Large White×Landrace crossbred male SPF pigs weighing ~16–20 kg were used in this experiment. The animals were acclimatized for two weeks and monitored by veterinarian for their general health status before the start of the experiment. During the experimental period, the pigs were maintained on commercial concentrate piglet feed. Water was provided ad libitum. Animal rooms were environmentally controlled with complete exchange of filtered air flow. The pigs were anesthetized by intravenous injection of Zoletil 50 (6 mg/kg body weight) during sample collection. Animals in severe conditions and at the end of the experiment were humanly euthanized using Zoletil 50 administered as 10 mg/kg body weight.

The pigs were randomly assigned into two groups and each group of pigs was kept in separate pens. Group A consisted of 5 pigs (A1–A5) was used to evaluate ASFV-SY18-∆CD2v/UK virulence and its protection. The pigs were injected intramuscularly (i.m.) with 1 mL of 10^4^ TCID_50_ ASFV-SY18-∆CD2v/UK. The dose used was determined based on the previous report for live attenuated ASFV [48]. Group B comprised 5 pigs (B1–B5) injected i.m. with PBS was used as a control group. After inoculation, the animals were monitored daily for any inappetence, water intake, and clinical sign of infection. Body temperature was monitored rectally. To monitor immune response, serum samples were collected on 7, 21, and 28 post-inoculation (dpi). To determine whether pigs develop viremia or shed the virus after inoculation with ASFV-SY18-∆CD2v/UK, whole blood and nasal swab samples were also collected on 21 and 28 dpi from the anesthetized pigs. Following inoculation, at 28 dpi, both groups of pigs were further i.m. challenged with 1 mL of 10^4^ TCID_50_ ASFV-SY18. The challenge dose was chosen based on previous related isolate virulence study [49]. After the challenge, all the animals were monitored daily for any respiratory symptoms, diarrhea, feed and water intake, and survival. Body temperature was measured rectally on daily basis. Serum, blood, and nasal swab samples were collected from the anesthetized pigs at 3, 6, 9, 12, 15, 18, and 21 days post-challenge (dpc). Following the challenge, samples were collected only from animals that did not exceed humane endpoints. Animals in severe conditions were euthanized. At the end of the experiment (21 dpc), all the surviving pigs were euthanized. Following euthanasia, a complete necropsy was performed, and tissue samples were collected from each animal.

#### 2.5.1. Detection of ASFV-Specific Antibodies

Serum samples were collected from the experimental pigs and the presence of ASFV-specific antibodies against the major capsid proteins, p72 and p30, were tested by competitive enzyme-linked immunosorbent assay (ELISA) according to manufacturer’s instructions (INGEZIM PPA Compac, Ingenasa, Madrid, Spain, and IDVet Innovative Diagnostics Louis Pasteur, Grabels, France). Briefly, all the reagents and samples were kept at room temperature before use. The diluent and sera, 50 µL of each, were incubated in wells coated with the p72 protein together with the positive and negative control samples. Samples and, positive and negative controls were prepared in duplicate. After subsequent washing, conjugate solution was added, incubated for 15 min and washed three times, and then the substrate solution was added. Finally, stop solution was added, and the plates were read at OD_450nm_. Similarly, p30 antigen coated plates were used to detect ant-p30 antibodies, and the assay was performed in duplicate following the manufacturer’s instructions.

#### 2.5.2. Real-Time PCR (qPCR)

Real-time PCR (qPCR) was conducted to detect ASFV-SY18-∆CD2v/UK and ASFV-SY18 genomes from the experimental pigs. DNA was extracted from the blood, nasal swab samples, and tissue homogenates taken from the experimental animals using AxyPrep nucleic acid isolation kits (Life Sciences, NY, USA), according the manufacturer’s protocols. ASFV genome was then detected using qPCR on the QuantStudio^TM^ system (Applied Biosystems, NY, USA) based on the OIE recommended primers [50]. The assay targets the highly conserved and antigenically stable C-terminal region of the p72 gene of ASFV and uses the 5′-nuclease assay (TaqMan^®^) system(Kumei Biotechnology Co., Ltd., Changchun, China) to detect the PCR amplification. To differentiate the mutant and parent ASFV genomes, qPCR targeting EGFP was designed, and samples collected after challenge were tested accordingly.

#### 2.5.3. Examination of Gross Lesions

Necropsy examination was done to assess any ASF-associated lesions with the euthanized pigs or dead pigs after the challenge. Briefly, macroscopic lesions in the thoracic and abdominal cavities, visceral organs, such as kidney, heart, liver, and lungs, and lymphoid tissues, such as spleen, mandibular, and inguinal lymph nodes, were examined in all the ASFV-SY18-∆CD2v/UK-inoculated and control pigs. During necropsy, tissue samples were also collected from each organ for ASFV genome detection.

### 2.6. Statistical Analysis

Graphs and statistical analyses of the virus kinetics, rectal temperature, and survival of animals were performed using GraphPad Prism (v8.2) software (GraphPad Software Inc., La Jolla, CA, USA). The normality of the data was tested using the Shapiro-Wilk test. Unpaired two-tailed Student’s *t*-test with equal variance [51], computed using GraphPad Prism, was used to analyze the significance of the virus titers difference between the mutant and wild-type ASFV. A two-way analysis of variance was used to compare the temperature means between groups. Kaplan-Meier survival procedure was used to compute the survival rate of the animals [52]. Differences with *p* < 0.05 were considered statistically significant. In all bar graphs, data are represented as average ± standard deviation.

## 3. Results

### 3.1. Generation of CD2v/UK Gene-Deleted ASFV-SY18

**Figure 1:**
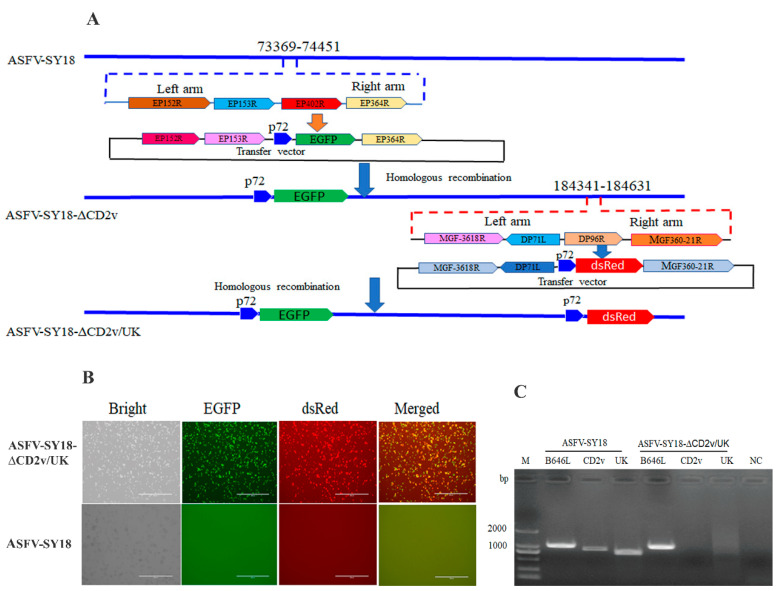
Deletion of the *CD2v* and *UK* genes from the ASFV-SY18 genome. (**A**) Schematic diagram representing the target site (s), the genes adjacent to the deletion sites and step-by-step generation of the double gene-deleted ASFV. The two recombinant transfer vectors designed to sequentially delete the *CD2v* gene at nt 73,369-74,451 and the *UK* gene at nt 184,341-184,631 of the ASFV-SY18 genome are shown. (**B**) ASFV-SY18-∆CD2v/UK amplification in alveolar macrophages (AM). ASFV-SY18-∆CD2v/UK- or ASFV-SY18-infected AM are shown in different fluorescence background (10×). (**C**) PCR analysis of ASFV-SY18-∆CD2v/UK stock. Viral DNA from ASFV-SY18-∆CD2v/UK or ASFV-SY18 infected AM were tested for the presence of *CD2v* or *UK* genes and the *B646L* (*p72*) gene was used as a positive control to detect ASFV genome, NC is a negative control.

In this study, a double gene-deleted ASFV-SY18 was generated through two rounds of successive homologous recombination. First, a CD2v-deleted ASFV (designated as ASFV-SY18-∆CD2v) was generated by homologous recombination between a recombinant transfer vector containing the *EGFP* gene (pOK12-p72CD2v-EGFP) and the parental ASFV-SY18 genome in AM as described above. The *CD2v* gene of 1,083 bp encoding about 361amino acid residues was replaced by an EGFP-expressing cassette. Then, the generated ASFV-SY18-∆CD2v was initially identified by an inverted fluorescent microscope (Nikon TE2000-U, Tokyo, Japan) observation (Figure 1A). The recombinant virus was purified on AM by the limiting dilution on 96-well plates, and recombinant virus clones were subsequently selected based on the homogenous EGFP fluorescence. ASFV-SY18-∆CD2v clones free of the parental virus containing EGFP were selected by PCR after 12 rounds of purification (Table 1), and the deletion of the CD2v-encoding gene and the insertion of *EGFP* were further confirmed by sequencing the deletion site.

In the second step of homologous recombination, ASFV-SY18-∆CD2v was then used as a backbone virus to generate a double gene-deleted virus and the *UK* gene, a 291 bp encoding ~85-97 amino acid residues, was replaced with the cassette containing *dsRed*. As described above, a homologous recombination between ASFV-SY18-∆CD2v and a recombinant transfer vector with the *dsRed* marker (pOK12-p72∆UK-dsRed) (Figure 1A) was used to generate ASFV-SY18-∆CD2v/UK. The generated recombinant virus, ASFV-SY18-∆CD2v/UK, was then initially identified based on the dsRed fluorescence background (Figure 1B), purified as described above, and, finally, confirmed by PCR (Figure 1C) for any parent virus presence targeting the *UK* gene. No *UK* gene amplicon was obtained, indicating that ASFV-SY18-∆CD2v/UK clone free of the parent virus was purified. The double gene-deleted virus stock was amplified in AM for downstream evaluation procedures.

### 3.2. In Vitro Replication and Hemadsorption of ASFV-SY18-∆CD2v/UK

**Figure 2:**
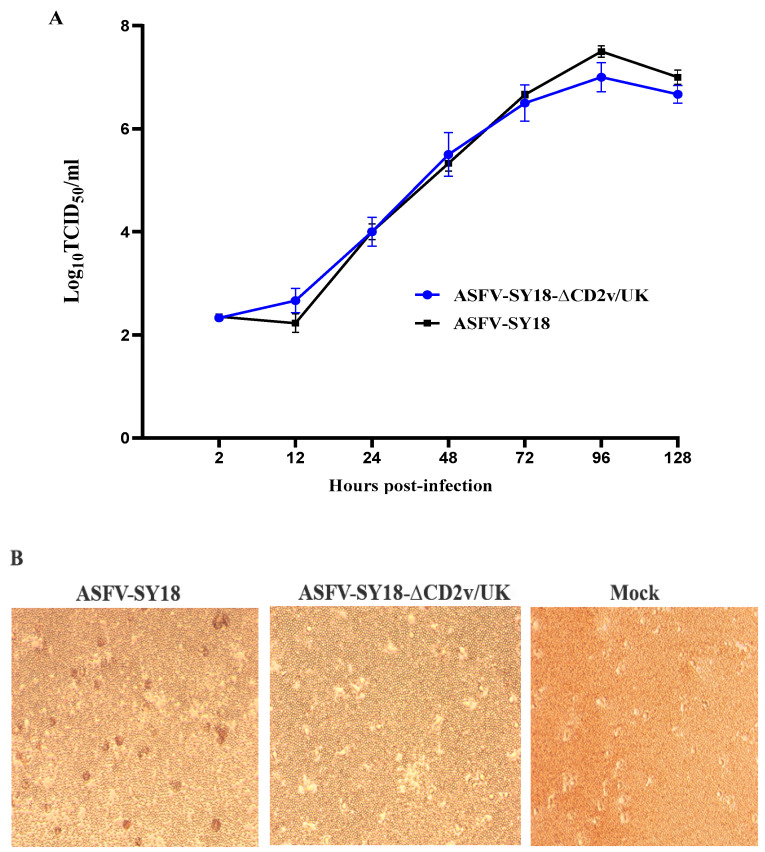
In vitro replication and hemadsorption of ASFV-SY18-∆CD2v/UK compared to the wild-type ASFV. (**A**) The in vitro replication kinetics of ASFV-SY18-∆CD2v/UK. The *y*-axis represents the virus titers recovered at different time points following infection to AM in TCID_50_/_mL_ on between 0 and 120 h post-infection. The data represent the means from two independent experiments and error bars represent standard deviations of the means. (**B**) The hemadsorbing activity of ASFV-SY18-∆CD2v/UK and wild-type ASFV in infected cells. Monolayers of AM infected with ASFV-SY18-∆CD2v/UK or wild-type ASFV or non-infected cells (Mock) were shown.

The in vitro growth kinetics of ASFV-SY18-∆CD2v/UK was evaluated in primary macrophage cultures, the primary targets of ASFV infection in pigs. The mutant and wild-type ASFVs were recovered from the infected AM at different hours post-infection (hpi) as described in Material and Methods. The titer of recovered viruses determined by fluorescence or HAD assay at each time point was used to construct the multistep growth curve. The results demonstrated that ASFV-SY18-∆CD2v/UK exhibited similar growth kinetics to the parent virus (Figure 2A). Therefore, the deletion *CD2v* and *UK* genes did not significantly reduce the ability of ASFV-SY18-∆CD2v/UK, relative to that of ASFV-SY18, to replicate in vitro in primary macrophage cultures. The results further suggest that ASFV-SY18-∆CD2v/UK amplification in AM would be feasible for practical use. Meanwhile, hemadsorbing characteristics was determined by adding porcine red blood cells to the virus-infected AM, and no “rosettes” of red blood cells were observed around ASFV-SY18-∆CD2v/UK-infected AM, while the parent virus-infected cells showed the hemadsorption, demonstrating that the CD2v deletion abolished the hemadsorbing activity of the virus (Figure 2B).

### 3.3. The Virulence of ASFV-SY18-∆CD2v/UK in Pigs

**Figure 3:**
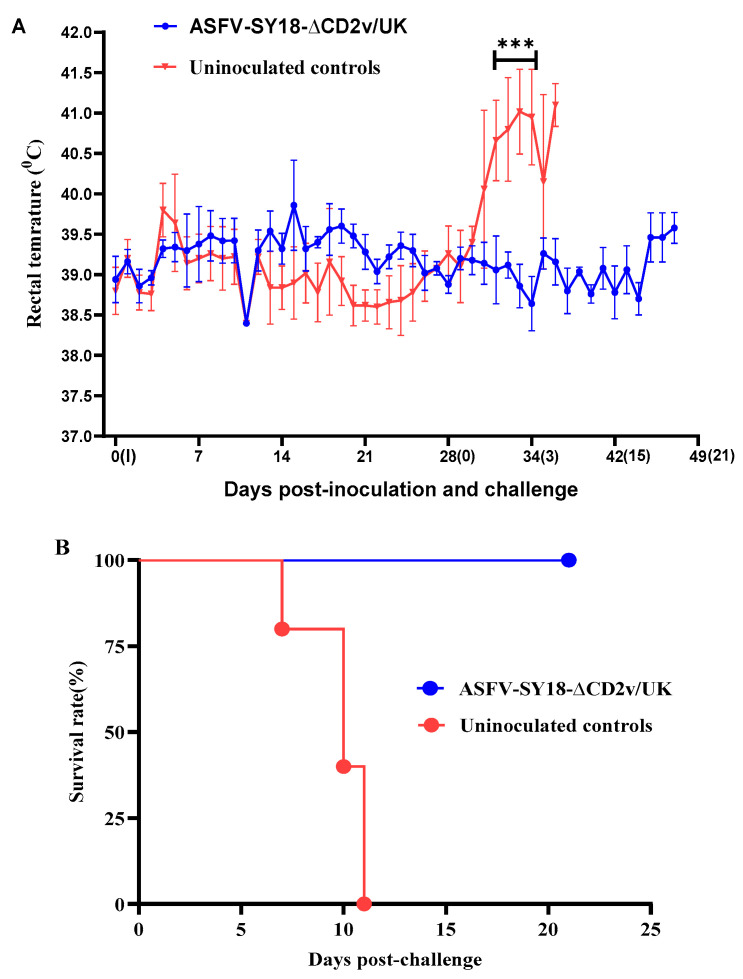
Rectal temperature and survival of the pigs following inoculation and challenge. (**A**) Rectal temperature (*y*-axis) for each group of pigs following inoculation and challenge. The date of inoculation (I) and challenge (0) are shown on the *x*-axis. Black asterisks indicate statistically significant differences between groups (** *p* < 0.001) and error bars represent the standard deviations. (**B**) Survival rate of the pigs inoculated with ASFV-SY18-∆CD2v/UK or control pigs after challenge with the parent virus.

To assess whether the deletion of the *CD2v* and *UK* genes affects the virulence of ASFV-SY18, pigs were i.m. inoculated with 1 mL of 10^4^ TCID_50_ ASFV-SY18-∆CD2v/UK. The animals i.m. inoculated with ASFV-SY18-∆CD2v/UK were monitored daily for 28 days. All the inoculated pigs did not display any clinical sign associated with ASF, indicating that ASFV-SY18-∆CD2v/UK become attenuated or less able to cause disease at the inoculated dose. The rectal temperature of the ASFV-SY18-∆CD2v/UK-inoculated pigs was in the normal range similar with the control group over the observation period (28 days) (Figure 3A). Therefore, deletion of the *CD2v* and *UK* genes resulted in attenuated ASFV-SY18. Pig A5 showed a transient higher temperature (40.1 °C) between 5 and 6 dpi. Pigs A1 and A4 also showed a slightly elevated rectal temperature (40.6 and 40.3 °C, respectively) at day 15. From the control group, 2 pigs (B1 and B3) showed a slightly higher temperature (40.7 and 40.1 °C, respectively) for one day (Figure 3A). The transient rectal temperature change in the inoculated or non-inoculated control pigs might not be associated with the inoculum.

The presence of virus genome was evaluated in blood and nasal swabs samples taken at 21 and 28 dpi by qPCR targeting the ASFV p72 gene [48]. No virus genome was detected in blood and nasal swab samples from any of the ASFV-SY18-∆CD2v/UK-inoculated pigs (group A) (Table 2 and Table 3). The results indicate that the virus could have been cleared, since the pigs did not show viremia or virus shedding.

### 3.4. Protective Efficacy of ASFV-SY18-∆CD2v/UK against ASFV-SY18 Challenge in Pigs

To evaluate whether ASFV-SY18-∆CD2v/UK induces protective immune responses, all the pigs (group A) were further challenged i.m. with 1 mL of 10^4^ TCID_50_ parental ASFV-SY18 at 28 dpi in parallel with the non-inoculated pigs (group B). All the ASFV-SY18-∆CD2v/UK-infected pigs remained healthy and displayed normal body temperature (Figure 3A), indicating that ASFV-SY18-∆CD2v/UK induced protective immune responses. On the contrary, all the ASFV-SY18 infected pigs in group B presented statistically significantly increase in body temperature starting from 3 dpc till the animals were euthanized (up to 41.5 °C), with respect to the pigs in group A, the ASFV-SY18-∆CD2v/UK-inoculated group (Figure 3A). All the pigs from the control group (group B) developed an acute form of ASF and were euthanized in severe conditions between 6 and 11 dpc. However, all the pigs belonging to group A survived the challenge, suggesting that the pigs have been protected due to pre-inoculation with ASFV-SY18-∆CD2v/UK (Figure 3B). The pigs in group A were euthanized at 21 dpc or at the termination of the experiment.

To assess the presence of virus genome in blood and nasal discharges after challenge with the parent virus, samples collected at 3, 6, 9, 15, 18, and 21 dpc from the two groups of pigs were tested by a real-time PCR targeting the *p72* gene. After challenge, a high level (CT = 22.4 ± 2.48) of ASFV DNA was detected in blood from all the control pigs (group B) (Table 2 and Table 3) at 6 dpc. In comparison, a low level (CT = 35.96 ± 0.35) of ASFV DNA was detected from pigs of group A (inoculated ASFV-SY18-∆CD2v/UK) in nasal swabs, and no ASFV DNA was detected in blood at 6 dpc, suggesting the challenge virus was not able to replicate to a high level in comparison to the control group. At the end of the experiment or when the animals died or were euthanized, tissue samples were collected from kidneys, livers, lungs, lymph nodes, and others. DNA extracted from the homogenized tissues was detected by real-time PCR as described above. A high-level of ASFV DNA (CT = 20.4 ± 6.9) was detected from the control group, for example, from submaxillary lymph nodes. In contrast, a low level of ASFV DNA (CT = 37 ± 1.1) was detected from lymphoid tissue of the pigs of group A. The low-level DNA from group A pigs further suggests that the challenge virus might not have been completely cleared (Table 4). Additionally, the samples collected from all the pigs after challenge were also tested using qPCR targeting *EGFP*, and all the samples were negative (data not shown here), further suggesting that the mutant virus was cleared.

### 3.5. Analysis of the Immune Response in ASFV-SY18-∆CD2v/UK-Inoculated Pigs

**Figure 4:**
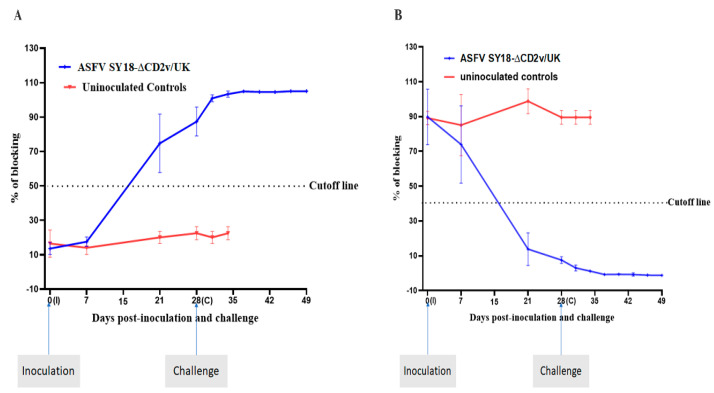
Detection of ASFV-specific antibodies in the pigs inoculated with ASFV-SY18-∆CD2v/UK. (**A**) Anti-p72 antibodies were detected using a blocking ELISA from the collected serum samples. Serum samples with a blocking percentage of ≥50% were considered positive. (**B**) Anti-p30 antibodies were detected using a blocking ELISA. Serum samples with a blocking percentage of ≤40% were considered positive. The data shown here is the mean of the blocking percentage of the animal per group on the indicated days. The error bars represent the standard deviations.

To evaluate the immune response, sera samples were collected at 0, 7, 21, and 28 dpi in parallel with the control group. The serum ASFV-specific antibodies were tested using two independent ELISA tests. As demonstrated in Figure 4A, in all the ASFV-SY18-∆CD2v/UK-inoculated pigs, anti-p72 antibodies were detected at 21 dpi. The blocking percentage for anti-p72 antibodies was 87.42 ± 8.43% at 28 dpi in the ASFV-SY18-∆CD2v/UK-inoculated pigs, while no antibody was detected in the control group. Similarly, ani-p30 antibodies were detected in all the inoculated pigs (Figure 4B). These results indicate that i.m. inoculated ASFV-SY18-∆CD2v/UK can induce an ASFV-specific humoral immune response. Following the challenge with the parent virus, sera samples were collected at 3, 6, 9, 12, 15, 18, and 21 dpc. The level of antibodies remained higher and increasing in the immunized pigs till the end of the experiment. In contrast, no ASFV-specific antibodies were detected in the control pigs, which were dead or euthanized between 6 and 11 dpc (Figure 4A,B).

### 3.6. Gross Pathological Lesions in the Inoculated Pigs Following the Challenge with ASFV-SY18

**Figure 5:**
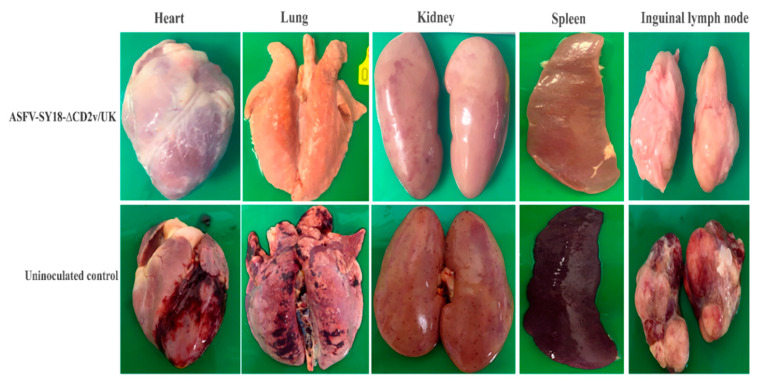
Macroscopic pathological lesions of the visceral organs and lymph nodes of pigs at necropsy.

At the end of the experiment, all the pigs were euthanized and examined for any gross lesion associated with ASF. The dead or euthanized control pigs were also assessed for any typical ASF gross lesions. All the pigs of group A did not show any gross lesions associated with ASF in their pericardial and peritoneal cavities, while the control group displayed hemorrhages both in pericardial and peritoneal cavities. The ASFV-SY-18-∆CD2v/UK-inoculated pigs (group A) did not show any ASF-associated gross lesions in their internal organs, such as liver, lungs, kidney, heart, brain, and spleen, as well as major lymph nodes, such as submaxillary, mandibular, and inguinal lymph nodes. This indicated that ASFV-SY-18-∆CD2v/UK was safe and protective. However, all the control pigs (group B) displayed the major gross lesions associated with the acute form of ASF in their internal organs. The heart displayed myocardial hemorrhage, the lungs were congested, kidneys developed multiple hemorrhagic spots, the spleen was enlarged, and the lymph nodes became enlarged and hemorrhagic (Figure 5).

## 4. Discussion

A vaccine against ASF has been a long-time priority to research. Unfortunately, there is no approved ASF vaccine. Only live attenuated ASF vaccines generated through gene-deletions are considered the most promising vaccines that would be achieved in the near future [23,53]. Various virulence-associated genes of ASFV have been identified [32,42,53,54], and, based on these pre-determined genetic factors, several specific gene-deleted ASFV isolates were generated, improving the empirical attenuation methods [37,38]. However, there exist inconsistent reports, and the associated safety issues remain major concerns [29,38,40]. Nonetheless, it would be possible to achieve the right balance between safety and immunogenicity. Safe and efficacious live attenuated ASF vaccines could most likely be obtained through serial deletions of virulence-determinant genes without affecting the in vivo viral replication and immunogenicity.

The ASFV EP402R ORF-encoded protein (CD2v) is responsible for selective adherence of red blood cells to ASFV-infected cells and for the association of extracellular virions with red blood cells [54]. Additionally, CD2v has a major role in inhibiting lymphocyte proliferation [39,55] and enhances replication of ASFV in the ticks [56]. Due to these stated important functions, *CD2v* has been considered as a genetic factor to possibly attenuate ASFV. For instance, EP402R-deleted Malawi Lil-20/1, a virulent isolate, was reported with a delayed onset of viremia [39]. The CD2v-deleted ASFV BA71 strain (BA71ΔCD2) has been shown with attenuated phenotype and capable of protecting pigs against homologous and heterologous strains [36]. In contrast, the *CD2v* gene-deleted Georgia 2007/1 has recently been shown to non-significantly affect the virulence [40]. The *UK* gene is a widely conserved gene across the different genotypes and has been reported as a nonessential gene for in vitro replication of ASFV. Moreover, the *UK* gene-deleted ASFV E70 strain exhibited reduced virulence and viremia in pigs [42], and the deletion of the *UK* and *9GL* genes altered the virulence of ASFV in pigs [38], demonstrating that the *UK* gene is a virulence determinant. However, both the BA71∆CD2v, and ASFV-G-Δ9GL/ΔUK remained at the experimental stage, like other MGF- and 9GL-deleted ASFV mutants [12,35,37,38]. Hence, the combined deletion of the *CD2v* and *UK* genes could attenuate the virulent strain and enhance the safety of the mutant virus.

In this study, we have successfully generated a double gene-deleted virus, ASFV-SY18-∆CD2v/UK, by series homologous recombination method and evaluate its phenotype, safety, and protection potential in pigs. The ASFV mutant remained stable at 20 passages, displaying similar in vitro replication with the parent virus and providing an important insight that the attenuated virus can be produced on large scale for future application. Our results are consistent with previous reports [36,40,42], suggesting that the *CD2v* and *UK* genes are non-determinants for in vitro ASFV replication. As expected, ASFV-SY18-∆CD2v/UK lost the ability to form “rosettes” of red blood cells around infected macrophages culture due to the *CD2v* deletion consistent with the previous reports [36,40]. More importantly, the ASFV-SY18-∆CD2v/UK-inoculated pigs remained clinically normal over the course of 28 days of observation, implying that the *CD2v/UK* genes deletion inactivated the pathogenicity of the virus in pigs. The findings of the current study are consistent with previous studies [36,42] and further confirm these genes are virulence-associated genes. Borca et al. [40], however, reported that the deletion of *CD2v* alone did not affect the in vivo phenotype of ASFV-G, a highly virulent virus related to ASFV-SY18 [10]. In this study, the simultaneous deletion of the two genes results in attenuated ASFV, which could be the direct outcome of the combined deletion of the two genes. Therefore, the simultaneous deletion of the two genes has attenuated the virus as expected. All the inoculated pigs were healthy over the observation period and developed an immune response. This suggests ASFV-SY18-∆CD2v/UK induces protective immune responses without causing disease. Moreover, no viral genome in the blood and nasal swab samples was detected in the ASFV-SY18-∆CD2v/UK-inoculated pigs, which could be the result of the development of host’s immune response, and, in turn, the animals could have cleared ASFV-SY18-∆CD2v/UK. Similar results were reported in pigs and wild boar vaccinated with MGF-deleted ASFV-G and Latvia in 2017, respectively, in which the attenuated virus was cleared by 28 days-post inoculation, and animals were tested negative as a result [48,57]. In separate studies it was demonstrated that the deletion of *CD2v* [36,39] and *UK* genes from different strains reduced the viremia in pigs [42]. In this study, the simultaneous deletion of the two genes has altered the virus virulence and its persistency in the blood. Furthermore, all the ASFV-SY18-∆CD2v/UK-inoculated pigs developed a strong ASFV-specific antibody response and were protected against the virulent parental strain challenge. These results suggest that ASFV-SY18-∆CD2v/UK has an optimal in vivo replication, which can stimulate the immune response without causing disease. However, ASFV DNA was detected delayed after challenge with weak positive PCR results from blood samples, as well as lymphoid tissues at necropsy. The results indicate that pigs might not have completely cleared the challenge virus, ASFV-SY18. This is an important issue and further research should be done in the future to determine whether the pigs shed infectious ASFV.

## 5. Conclusions

In conclusion, in this study, we generated a double-gene-deleted ASFV, ASFV-SY18-∆CD2v/UK, and characterized its in vitro replication and hemadsorption properties. More importantly, we determined the in vivo phenotypes and immune response, as well as protection potency. The results presented in this work indicate that the *CD2v* and *UK* genes deletion did not affect the in vitro replication of the virus. Moreover, ASFV-SY18-∆CD2v/UK was avirulent in vivo at the provided dose, and all the inoculated animals were protected against the virulent challenge. Further large-scale studies and clinical trials are warranted to verify our promising results. In future works, the efficacy of this proposed vaccine would be evaluated in animals under different physiological state and its long-term effects at different doses and administration routes.

## Figures and Tables

**Table 1:** Primers used in the study.

Name	Sequence (5′–3′)	Description
CD2vd-LA-F	GGTACCCGGGAGCTCGAATTCGTCTAGTTATATATGTCGGTCA	Used to amplify the left arm
CD2vd-LA-R	GACTTTTCTCCGGCGACCCTTTATGAACATATGTTTTATA	
CD2vd-m-F	TATAAAACATATGTTCATAAAGGGTCGCCGGAGAAAAGTC	Used to amplify the EGFP marker gene
CD2vd-m-R	GGTTAAATAATTAATATATAGTTATCTAGATCCGGTGGATCCC	
CD2vd-RA-F	GGGATCCACCGGATCTAGATAACTATATATTAATTATTTAACC	Used to amplify the right arm
CD2vd-RA-R	GTCTGCAGAAGCTTCGAATTCGATATTTTGGCTATCATCGG	
CD2v-F	CACTAGCTACATGTGGAAAAGCAGG	Used to detect wild-type ASFV in CD2v-deleted ASFV purification
CD2v-R	GGGTAGATAATGGCGGGATATTG	
UKd-LA-F	CCCGGGAGCTCGAATTCGAAGCTTTTCACCTTTATGAAATGATC	Used to amplify the left arm
UKd-LA-R	GACTTTTCTCCGGCGACCCGCTAATAGTTACTATACAAAAATAG	
UKd-m-F	CTATTTTTGTATAGTAACTATTAGCGGGTCGCCGGAGAAAAGTC	Used to amplify the dsRed marker gene
UKd-m-R	GGATGGAGCGCATTAGGGATTACAGGAACAGGTGGTGGC	
UKd-RA-F	GCCACCACCTGTTCCTGTAATCCCTAATGCGCTCCATCC	Used to amplify the right arm
UKd-RA-R	GCCACGGCGATATCGGATCCAATAGAGTCATTATTTAATAATAGG	
UK-F	CCGCCTCCCCATTATTCTTC	Used to detect wild-type ASFV in UK-deleted ASFV purification
UK-R	GATGGAGCGCATTAGGGATCCC	
p72-F	GCTCGCCGAAGGGAATGGATAC	Used to detect ASFV DNA in recombinant virus isolation
p72-R	GGCCGACAAGATTATATTGG	
p72-F	CTGCTCATGGTATCAATCTTATCGA	Used in real-time PCR
p72-R	GATACCACAAGATCAGGCCGT	
eGFP-F	AGTCCGCCCTGAGCAAAGA	Used in real-time PCR to detect EGFP
eGFP-R	TCCAGCAGGACCATGTGATC	

(F) forward; (R) reverse; (RA) right arm; (LA) left arm; (m) marker; (d) deletion.

**Table 2:** Detection of ASFV genome in the blood of the experimental pigs following inoculation and challenge.

Group	CT Value of Real-Time PCR										
		Days Post-Inoculation			Days Post-Challenge						
	No.	0	21	28	3	6	9	12	15	18	21
	A1	N	N	N	N	N	N	N	N	36.50	36.60
ASFV-SY18-∆CD2v/UK	A2	N	N	N	N	N	N	N	N	36.90	37.10
	A3	N	N	N	N	N	N	N	N	36.70	36.80
	A4	N	N	N	N	N	N	N	N	37.01	37.06
	A5	N	N	N	N	N	N	N	N	36.92	36.78
	B1	N	N	N	25.6	18.0	/				
PBS	B2	N	N	N	26.7	23.4	/				
	B3	N	N	N	26.9	23.1	21.4	/			
	B4	N	N	N	26.7	23.4	/				
	B5	N	N	N	26.9	24.1	21.4	/			

(N) no CT value; (/) died or euthanized.

**Table 3:** Detection of ASFV genome in the nasal swab samples from the pigs following inoculation and challenge.

	CT Values of Real-Time PCR										
Group		Days Post-Inoculation			Days post-challenge						
	No.	0	21	28	3	6	9	12	15	18	21
	A1	N	N	N	N	35.70	36.80	35.40	37.90	36.60	36.80
	A2	N	N	N	N	36.10	36.80	36.90	37.10	35.60	36.20
ASFV-SY18-∆CD2v/UK	A3	N	N	N	N	36.40	35.80	37.10	38.00	37.76	37.80
	A4	N	N	N	N	35.50	35.30	36.30	36.10	39.52	39.00
	A5	N	N	N	N	36.10	37.20	35.10	35.40	35.83	36.00
	B1	N	N	N	36.40	21.00	/				
	B2	N	N	N	N	35.00	28.10	/			
PBS	B3	N	N	N	33.50	23.00	22.70	/			
	B4	N	N	N	33.10	/					
	B5	N	N	N	33.40	/					

(N) no CT value; (/) died or euthanized.

**Table 4:** Detection of ASFV genome in organs and lymph nodes of pigs following the challenge with ASFV-SY18.

Group	CT Value of Real-time PCR										
	No.	Heart	Lung	Spleen	Liver	Kidney	Bladder	Ig. ln	Ms. ln	Tonsil	Sub. m ln
ASFV-SY18- ∆CD2v/UK	A1	N	N	N	N	N	N	36.50	N	36.70	37.50
	A2	N	N	N	N	N	N	N	N	37.20	36.80
	A3	N	N	N	N	N	N	N	N	N	39.19
	A4	N	N	N	N	N	N	36.73	37.80	36.48	36.29
	A5	N	N	N	N	N	N	N	N	37.20	37.10
PBS	B1	16.50	18.90	25.40	18.70	23.50	27.00	17.20	19.50	22.00	19.00
	B2	25.00	23.00	22.80	18.90	26.50	24.80	26.00	22.00	22.00	24.00
	B3	24.80	16.20	23.80	19.30	21.00	25.50	22.00	20.70	22.20	22.20
	B4	18.33	18.70	17.50	21.50	19.00	27.00	18.40	20.50	23.00	15.00
	B5	19.30	19.70	25.20	20.90	18.94	24.80	24.77	20.50	22.70	22.00

(In. ln) inguinal lymph node; (Ms. Ln) mesenteric lymph node; (Sub. m. ln) submaxillary lymph node; (N) no CT value.
